# Faster 3D saturation-recovery based myocardial T1 mapping using a reduced number of saturation points and denoising

**DOI:** 10.1371/journal.pone.0221071

**Published:** 2020-04-10

**Authors:** Giovanna Nordio, Aurelien Bustin, Freddy Odille, Torben Schneider, Markus Henningsson, Claudia Prieto, René M. Botnar

**Affiliations:** 1 School of Biomedical Engineering and Imaging Sciences, King’s College London, London, England, United Kingdom; 2 CIC-IT 1433, INSERM, Université de Lorraine and CHRU de Nancy, Nancy, France; 3 IADI, INSERM U1254 and Université de Lorraine, Nancy, France; 4 Philips Healthcare, Guildford, England, United Kingdom; 5 Escuela de Ingeniería, Pontificia Universidad Católica de Chile, Santiago, Chile; New York University School of Medicine, UNITED STATES

## Abstract

**Purpose:**

To accelerate the acquisition of free-breathing 3D saturation-recovery-based (SASHA) myocardial T1 mapping by acquiring fewer saturation points in combination with a post-processing 3D denoising technique to maintain high accuracy and precision.

**Methods:**

3D SASHA T1 mapping acquires nine T1-weighted images along the saturation recovery curve, resulting in long acquisition times. In this work, we propose to accelerate conventional cardiac T1 mapping by reducing the number of saturation points. High T1 accuracy and low standard deviation (as a surrogate for precision) is maintained by applying a 3D denoising technique to the T1-weighted images prior to pixel-wise T1 fitting. The proposed approach was evaluated on a T1 phantom and 20 healthy subjects, by varying the number of T1-weighted images acquired between three and nine, both prospectively and retrospectively. Following the results from the healthy subjects, three patients with suspected cardiovascular disease were acquired using five T1-weighted images. T1 accuracy and precision was determined for all the acquisitions before and after denoising.

**Results:**

In the T1 phantom, no statistical difference was found in terms of accuracy and precision for the different number of T1-weighted images before or after denoising (P = 0.99 and P = 0.99 for accuracy, P = 0.64 and P = 0.42 for precision, respectively). In vivo, both prospectively and retrospectively, the precision improved considerably with the number of T1-weighted images employed before denoising (*P*<0.05) but was independent on the number of T1-weighted images after denoising.

**Conclusion:**

We demonstrate the feasibility of accelerating 3D SASHA T1 mapping by reducing the number of acquired T1-weighted images in combination with an efficient 3D denoising, without affecting accuracy and precision of T1 values.

## Introduction

Late gadolinium enhancement (LGE) is the reference technique for the visualization of myocardial scar and focal fibrosis, however it cannot visualize diffuse fibrosis [1]. To identify diffuse fibrosis, myocardial T1 mapping has been proposed as a powerful alternative approach. In addition, T1 mapping in combination with a gadolinium-based contrast agent and with the knowledge of the blood haematocrit value permits to assess the extracellular volume (ECV), which has been shown to be in good agreement with collagen deposition [2]. Several myocardial T1 mapping techniques have been proposed and are based on the acquisition of multiple T1-weighted images along the recovery curve of the magnetization after an initial inversion recovery [3], saturation recovery [4] or a combination of both inversion and saturation recovery [5]. The modified Lock-Locker inversion recovery (MOLLI) [3] technique is the most widely used T1 mapping approach, and involves acquiring a single 2D T1 map during a breath-hold [3] or multiple 2D slices during free-breathing [6]. MOLLI is highly reproducible and provides precise myocardial T1 maps, however it underestimates T1 due to the acquisition of multiple inversion recovery images spaced over multiple heartbeats after a single inversion pulse, and T2 and magnetization transfer (MT) dependency [7,8]. An alternative 2D saturation recovery single-shot (2D SASHA) technique, employs a saturation-recovery pulse followed by the acquisition of one T1-weighted image in the same heartbeat, which is then repeated with varying saturation delays to sample the entire saturation recovery curve and subsequently generate the myocardial T1 map [4]. SASHA permits to obtain more accurate and heart-rate independent T1 maps than MOLLI but its precision is lower, due to the smaller dynamic range compared to inversion recovery based techniques [8,9]. Recently, we demonstrated the feasibility of a free-breathing 3D SASHA [10] imaging technique, which allows to provide whole-heart coverage with higher signal-to-noise ratio (SNR) and image resolution than with conventional 2D approaches. We have also demonstrated that the precision of 3D SASHA can be improved, without affecting the T1 accuracy, using a post-processing 3D denoising technique based on Beltrami regularization applied directly on the T1-weighted images [11]. The acquisition time of the 3D SASHA sequence is considerably longer than a breath-hold (in the order of 12 min), thus 1D diaphragmatic navigator gating (and tracking) was employed to enable 3D free-breathing T1 mapping [10].

2D and especially 3D T1 mapping techniques suffer from long acquisition times due to the need of acquiring several images along the inversion or saturation recovery curve. Different sampling schemes along the recovery curve have been proposed for 2D MOLLI in order to reduce the number of heartbeats and thus shorten the length of the breath-hold, reducing the total number of heartbeats per breath-hold from 17 to 9 [12,13]. Undersampling reconstruction techniques have also been investigated to accelerate the acquisition and improve the image resolution of T1 maps [14–16]. Simultaneous and interleaved multi-slice imaging techniques have been also investigated, both in free-breathing and under breath-hold, in order to accelerate the acquisition and to increase volume coverage [6,17]. However, these approaches suffer from limited spatial resolution and do not provide whole-heart coverage.

In this study, we propose to accelerate the 3D SASHA acquisition by reducing the number of the T1-weighted images acquired along the saturation recovery curve. To overcome the expected loss in accuracy and precision due to the reduced number of T1-weighted images acquired, we use the 3D denoising technique based on Beltrami regularization applied directly to the T1-weighted images prior to T1 fitting (as is [11]), enabling accurate and precise 3D SASHA T1 mapping from fewer saturation points and thus shorter scan times. The proposed approach was tested on a standardized T1 phantom, 10 healthy subjects with retrospectively reduced number of T1-weighted images, 10 healthy subjects with prospectively varied number of T1-weighted images and three patients with suspected cardiovascular disease.

## Materials and methods

### Imaging sequence

**Fig 1:**
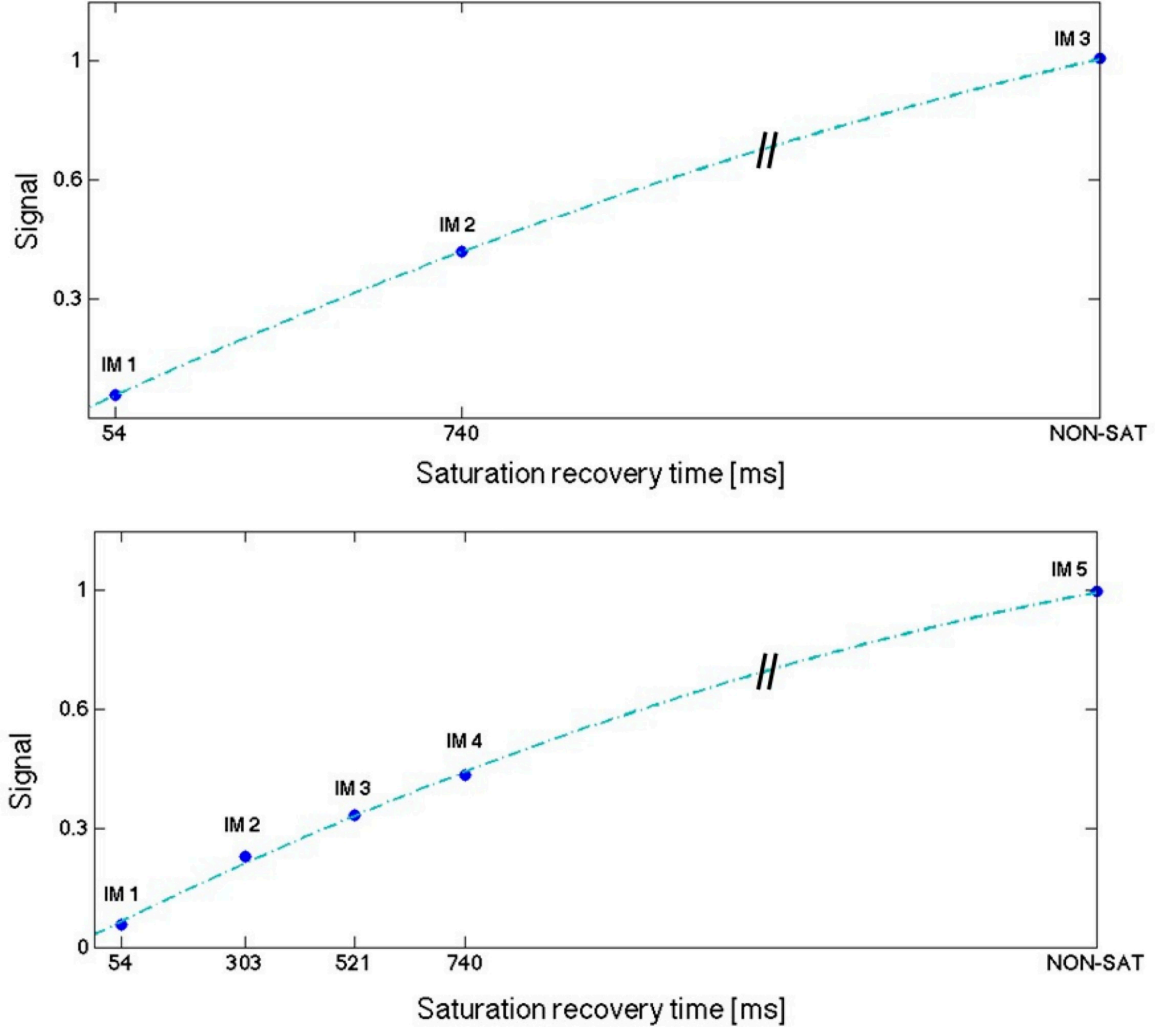
Distribution of three (A) and five (B) time points along the T1 recovery curve.

The 2D SASHA T1 mapping sequence involves acquiring eleven T1-weighted images at different saturation points [4]. The original 3D SASHA T1 mapping sequence [10], as well as its version with improved precision [11], acquires and fits nine T1-weighted images along the T1 recovery curve. In this study, the number of images acquired along the saturation recovery curve with the 3D SASHA sequence was varied between three and nine in order to investigate the effect of this parameter on the accuracy and precision of the T1 maps. Fig 1 shows the distribution of three and five time points along the T1 recovery curve used in this study. An image without any saturation preparation was acquired at the beginning of the scan, which corresponds to a measurement of the fully recovered magnetization (last point on the graphs). The saturation time points were then acquired with equal distribution between the minimum and the maximum saturation time (54 ms and 740 ms, respectively, for a heart rate of 60 bpm), following the original implementation of the 2D SASHA [4] imaging sequence.

### 3D denoising

A reduced number of T1-weighted images is expected to decrease the precision of the corresponding T1 maps, which could result in noisy T1 maps. In order to achieve high precision in spite of the reduced number of saturation recovery time points we apply the 3D Beltrami denoising technique, previously presented in [11], to the T1-weighted images prior to the T1 fitting. The Beltrami framework for image denoising and enhancement was introduced for 2D natural images by Sochen, Kimmel and Malladi [18], proposed for 2D myocardial T1 mapping denoising by Bustin et al [19] and have been recently extended to 3D T1 mapping denoising [11]. The Beltrami regularization allows to preserve the edges, while reducing the noise of the images without introducing staircasing artefacts [20]. The Beltrami denoising method was compared against non-local means (NLM) denoising for 2D T1 mapping, and it was found to be significantly better than NLM in terms of image quality (signal-to-noise ratio), accuracy (mean T1) and precision (standard deviation of T1 as a surrogate for precision) for T1 value estimation (19).

In this work, the 3D Beltrami denoising framework is applied to 3D T1 mapping with reduced number of time points, by exploiting the redundant information in both the 3D spatial and T1 recovery dimensions, to accelerate the acquisition.

### Imaging

The proposed approach was evaluated in a standardized phantom, 20 healthy subjects and three patients with suspected cardiovascular disease. All imaging studies were performed on a 1.5T MR scanner (Ingenia, Philips, Best, The Netherlands). The study was performed in accordance with the Declaration of Helsinki (2000) and was approved by the Institutional Review Board (IRB) at St Thomas’ Hospital. All subjects recruited to this study provided written informed consent with study approval from the IRB (1/11/12 and 15/NS/0030).

### Phantom study

A standardized T1 phantom with nine agar/NiCl2 vials was used for imaging, with T1 values in the range of 250 to 1500 ms [21]. The phantom was imaged using the original 3D SASHA sequence, with nine images acquired at different saturation points (54–650 ms) in two different scanning sessions. The nominal scan time for the 3D SASHA sequence was of 4:14 minutes:seconds. The number of T1-weighted images for the T1 fitting was reduced retrospectively from nine to three (step size of one). For each case, T1 maps were obtained using a three parameter-fitting model before and after 3D denoising. An inversion recovery spin-echo (IRSE) sequence was used as reference method for the T1 values of the vials. The acquisition parameters used for the 3D SASHA sequence were: FOV = 300 x 300 x 90 mm^3^, image resolution = 1.4 x 1.4 mm^2^, slice thickness = 8 mm, flip angle = 35°, TR/TE = 3.3/1.6 ms, parallel imaging with a SENSE factor of 2 in the phase-encoding direction, balanced steady-state free precession (bSSFP) acquisition. Sequence parameters for the spin-echo sequence were: FOV = 200 x 200 mm^2^, image resolution = 3.1 x 3.1 mm^2^, 10 mm slice thickness, TR = 8000 ms and TE = 5.9 ms. The phantom was acquired with a simulated heart rate of 60bpm and using a combination of the 12-channel posterior and 16-channel anterior torso coils.

### Healthy subject study

The effect of reducing the number of T1-weighted images acquired along the recovery curve was studied in healthy subjects retrospectively and prospectively. For the retrospective study, data was collected in 10 healthy subjects using the original 3D SASHA sequence with nine images acquired at different saturation points. The number of T1-weighted images for the T1 fitting was modified retrospectively from three to nine (step size of one) and mapping was performed before and after denoising. For the prospective study, data was acquired in 10 additional healthy subjects using the 3D SASHA sequence with three, five and nine T1-weighted images along the recovery curve. The acquisition parameters used for the 3D SASHA sequence (both retrospective and prospective studies) were: FOV = 300 x 300 x 90 mm^3^, image resolution = 1.4 x 1.4 mm^2^, slice thickness = 8 mm, flip angle = 35°, TR/TE = 3.3/1.6 ms, subject specific mid-diastolic trigger delay and saturation times, short-axis orientation, and 32-channel coil. 1D diaphragmatic navigator gating and tracking was used for respiratory motion compensation with a 5mm end-expiratory gating window. For both prospective and retrospective studies, the 3D SASHA T1 map with nine images along the recovery curve was considered as the reference standard. 3D SASHA with nine images along the recovery curve has been previously compared against 2D MOLLI showing excellent agreement in terms of accuracy [11].

### Patient study

The feasibility of using a reduced number of T1-weighted images was investigated in patients using five saturation points along the recovery curve. The number of saturation points for the prospective patient scan was set conservatively based on the results obtained in healthy subjects. 3D SASHA was acquired before contrast injection in three patients with suspected cardiovascular disease with the same parameters used in the healthy subject study but with a lower in plane resolution of 1.6 x 1.6 mm^2^ to further reduce scan time. Late gadolinium enhancement images were acquired 10–20 minutes after injection of 0.1–0.2 mmol/kg of Gadovist and used as ground truth to determine the presence of fibrosis. The acquisition parameters used for the 2D LGE sequence include: FOV = 350 x 350 mm^2^, in plane resolution of 1.6 x 1.9 mm^2^, slice thickness = 10 mm, flip angle = 25°, TR/TE = 6.1/3 ms.

### Image analysis

3D denoising and three-parameter fitting were performed offline using MATLAB (MathWorks, Natick, MA). A T1 map was obtained for each acquisition, using different number of T1-weighted images, both before and after applying the 3D denoising method. A region of interest (ROI) was manually drawn in each phantom vial and in the septum of the myocardium for the mid slice T1 map in all subjects. Mean and standard deviation in the selected ROIs were calculated and used as a measurement of accuracy and surrogate for precision of the T1 map. The measurements were compared using a Kruskal-Wallis test to identify if there was any significant difference between acquisitions with different numbers of T1-weighted images and between corresponding non-denoised and denoised T1 maps. Both for the in vivo retrospective and prospective study, intensity profiles across the blood and the myocardium have been compared before and after denoising in order to evaluate the effect of the denoising method on the T1maps. Repeatability of the T1 values measured for each vial between the two scanning sessions was quantified. For statistical analysis, GraphPad Prism v5 for Windows (GraphPad Software, La Jolla, CA) was used with a threshold of *P* < 0.05 to define the statistical significance. For the in vivo prospective study, the image quality of the 3D SASHA T1 maps acquired with three, five and nine images were assessed by a cardiologist (R.H., 2 y of experience in cardiac MRI, SCMR level 2). The reader was blinded to the acquisition type and he was asked to grade the image quality of the T1 maps on a 4-point scoring system: 1 for non-diagnostic images, 2 for poor image quality images, 3 for acceptable image quality, and 4 for fully diagnostic images. A Bland-Altman plot was calculated in order to compare the accuracy and the precision of the 3D SASHA non-denoised vs. 3D SASHA denoised, both for the retrospective and prospective study. In addition, for the prospective study, an AHA segmentation [22] was calculated to compare the mean and standard deviations (spatial uniformity) measured in the different myocardial segments of the whole-heart 3D SASHA for different number of saturation points before and after denoising. This segmentation enables the analysis of the spatial homogeneity of the accuracy and precision for a different number of saturation time points before and after denoising. Three slices (apex, mid and base) and 16 segments were used to represent the cardiac volume, while the 17^th^ segment represented the blood pool.

## Results

### Phantom study

There was good agreement in terms of accuracy between the original 3D SASHA imaging sequence with nine T1-weighted images and the gold standard IRSE sequence, both before and after denoising, as shown in the Bland Altman plot in S1 Fig.

**Fig 2:**
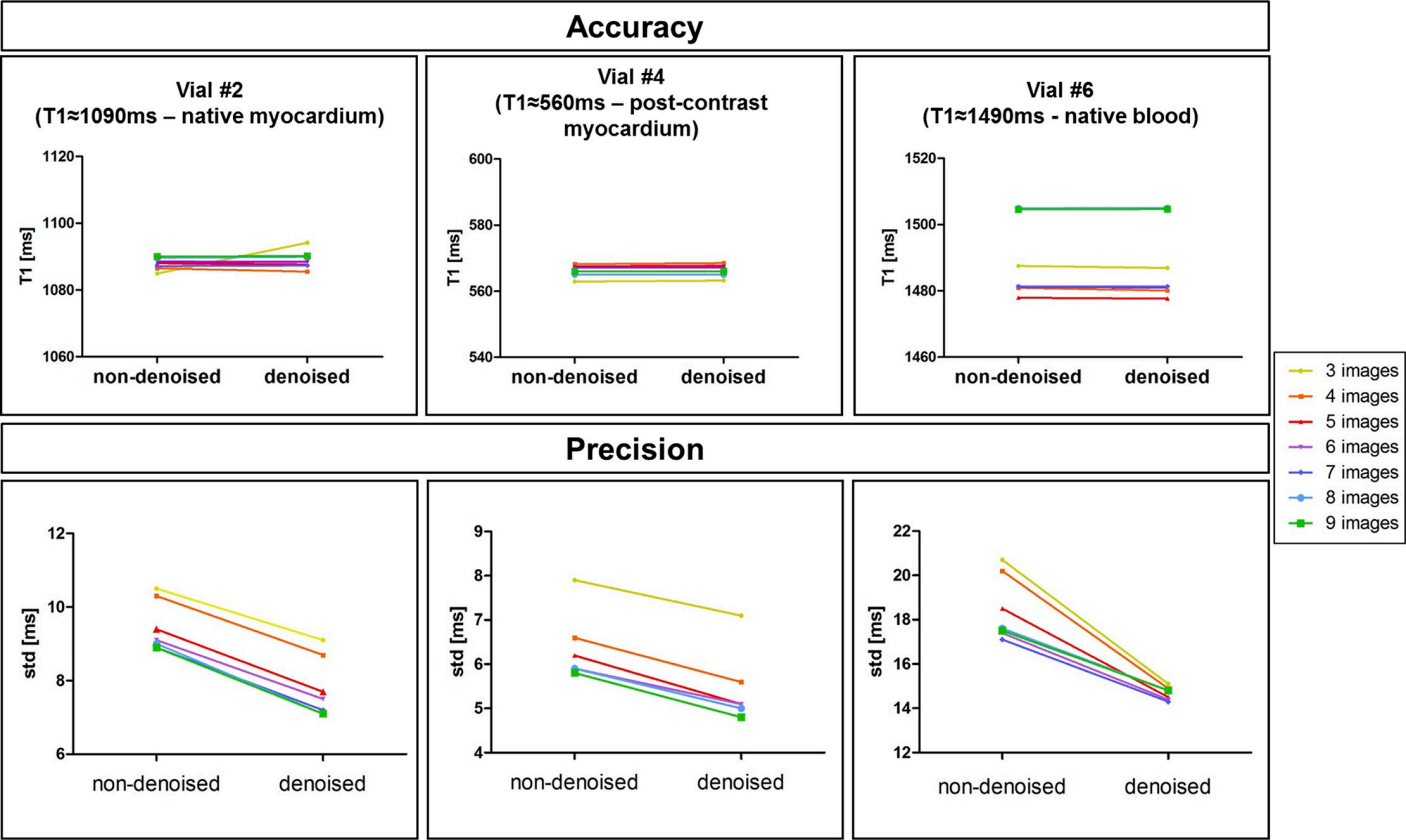
Accuracy and precision of three representative vials of the T1 phantom (native myocardium, post-contrast myocardium, native blood), measured before and after 3D denoising. The number of T1-weighted images used to generate the T1 maps was modified retrospectively from three to nine (step size of one), indicated by the different colors.

**Fig 3:**
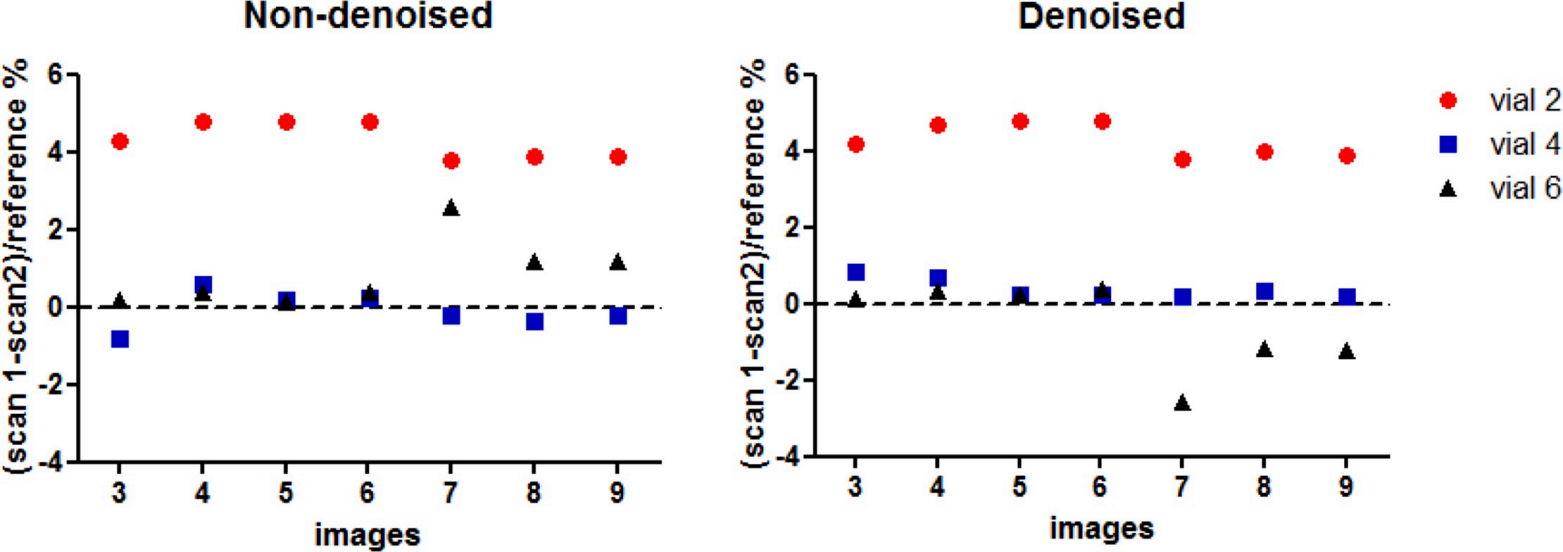
Repeatability of T1 values measured on the vials 2 (T1 = 1078ms), 4 (T1 = 559ms) and 6 (T1 = 1507ms) between scans performed in two separated sessions. 3D SASHA technique was acquired with 9 saturated images, and retrospectively reconstructed with 3 to 9 images before and after denoising. Repeatability with denoised and non-denoised 3D SASHA for all three vials is comparable.

Accuracy and precision measured in three phantom vials are shown in Fig 2, with T1 values similar to healthy native myocardium (vial #2), post-contrast myocardium (vial #4) and native blood (vial #6), and for different numbers of T1-weighted images in comparison to the gold standard spin echo values, indicated for each vial in Fig 2. For all the vials, there was no significant difference in terms of accuracy for the different number of T1-weighted images before or after denoising (respectively P = 0.99 and P = 0.99), as well as in terms of precision before or after denoising (respectively *P* = 0.64 and *P* = 0.42). The nominal scan time for the 3D SASHA sequence with three images acquired along the recovery curve was 1min 25s. The repeatability of the T1 values, measured on vials 2,4 and 6, before and after denoising, between scans performed in two different sessions is shown in Fig 3. For vial 4 and 6, which have a T1 similar to post-contrast myocardium and blood, the variation in T1 between the two scans is less than 1%, while for the vial 2 with T1 similar to pre-contrast myocardium is of 4.3%.

### Healthy subjects study

#### Retrospective study

**Fig 4:**
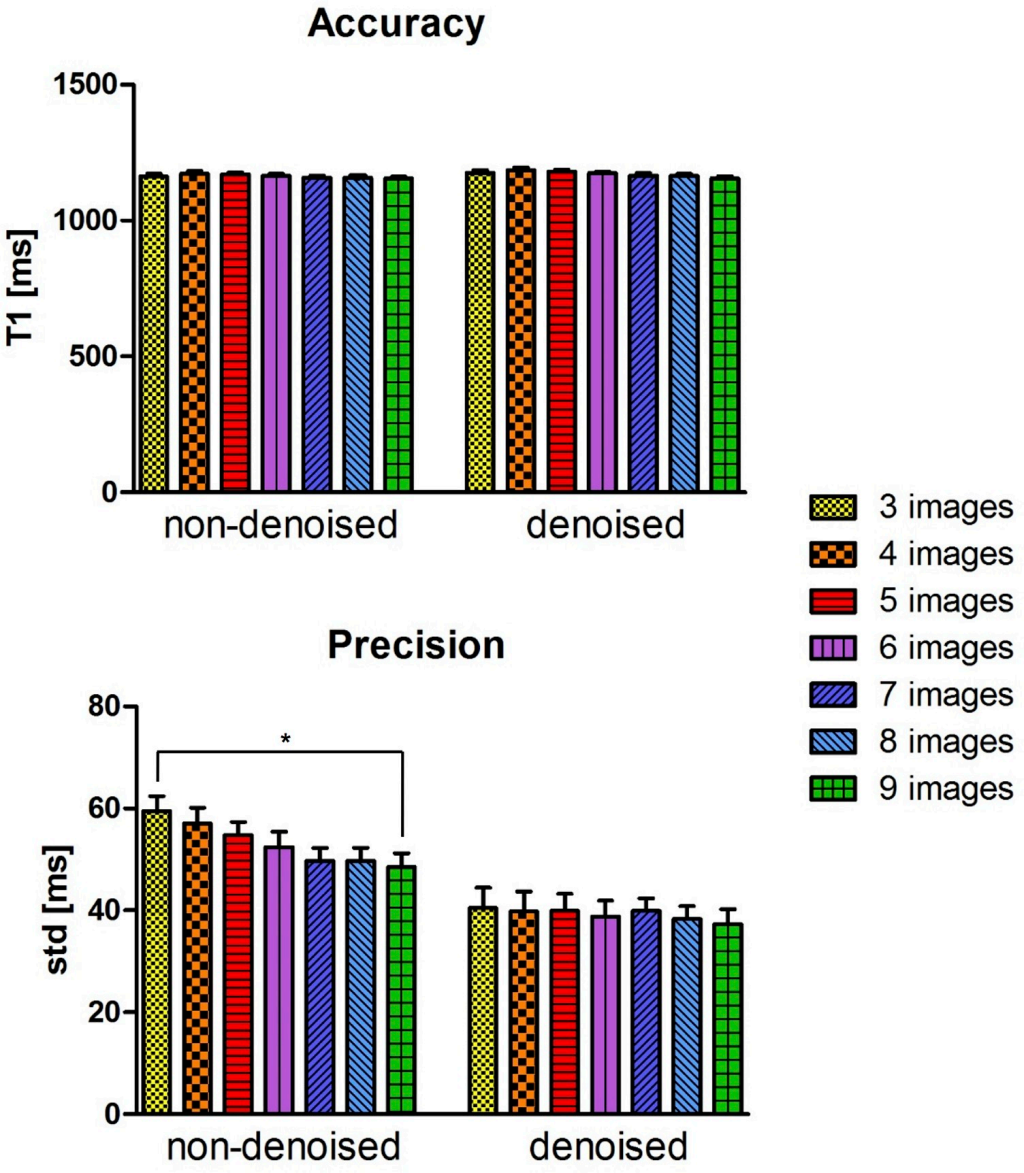
Accuracy and precision averaged between the ten healthy subjects for the retrospective study (3 to 9 images considered for T1 mapping). A ROI was manually drawn in the septum of the myocardium in the mid slice of the 3D SASHA T1 maps before (non-denoised) and after (denoised) denoising. Statistical significance difference is indicated by * (p value < 0.045).

**Fig 5:**
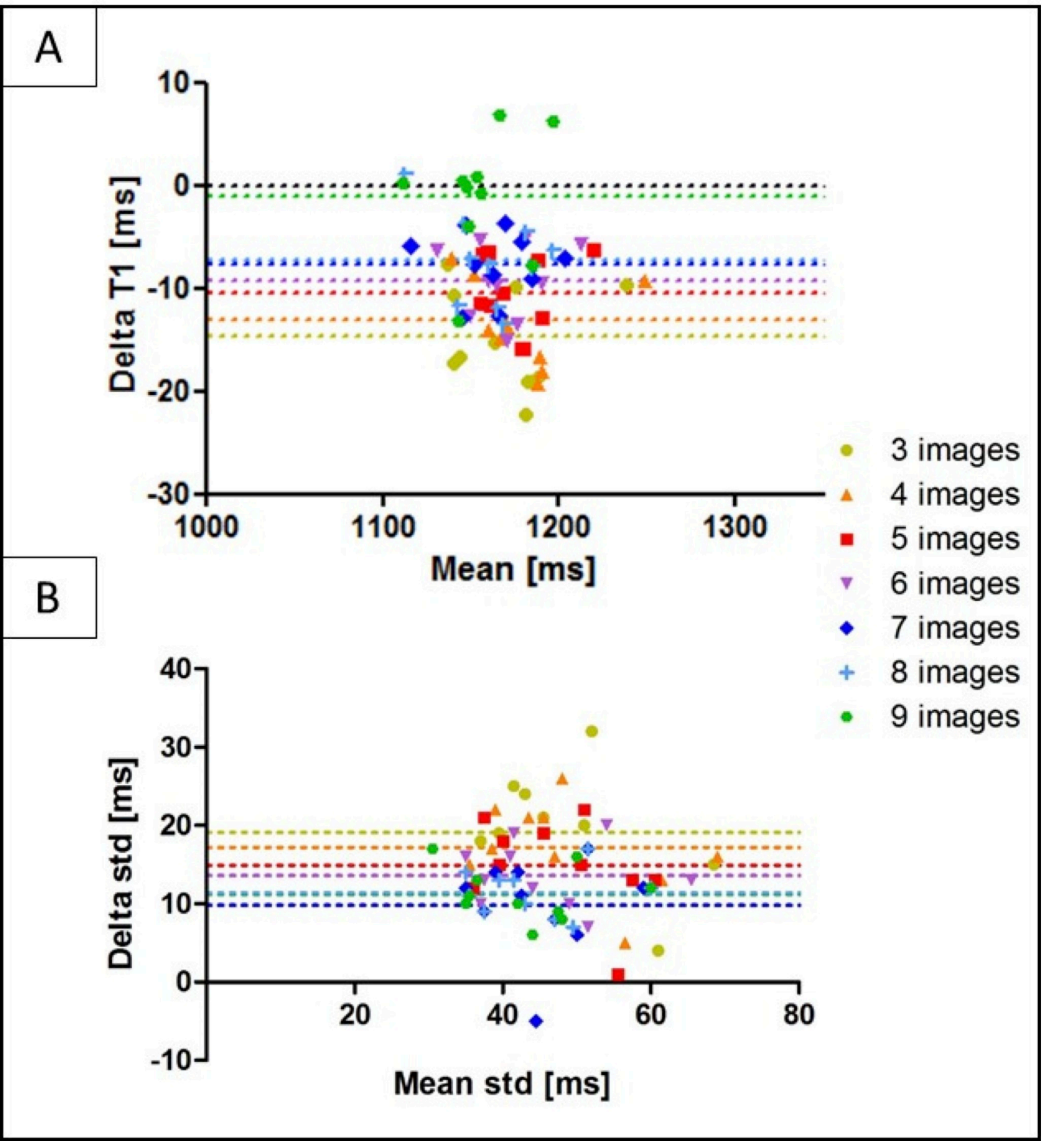
Bland-Altman plot of the accuracy (A) and precision (B) of the non-denoised vs. denoised 3D SASHA using three to nine T1-weighted images for imaging, indicated with different colors in the figure. The x-axis shows the mean between the measurements of the non-denoised and denoised 3D SASHA, while the y-axis shows the difference (delta T1) between the two measurements. The bias of each measurement is also indicated in the plot.

The accuracy and precision averaged over all ten healthy subjects for the retrospective study (three to nine images considered for mapping) both before and after denoising is shown in Fig 4. There was no statistical significant difference between the accuracy measured on the T1 maps reconstructed with three compared to nine T1-weighted images, both before and after denoising (respectively *P* = 0.48 and *P* = 0.14). There was a statistical difference (*P* < 0.05) between the precision measured on the T1 maps reconstructed with three and nine T1-weighted images before denoising, while there was no statistical difference after denoising (*P* = 0.99). There was no statistical difference between the precision measured on the T1 maps reconstructed with four to nine T1-weighted images, both before and after denoising (*P* = 0.99). The agreement in terms of accuracy and precision between the non-denoised and denoised 3D SASHA T1 maps obtained using from three to nine T1-weighted images is shown in Fig 5 through Bland-Altman plots.

**Fig 6:**
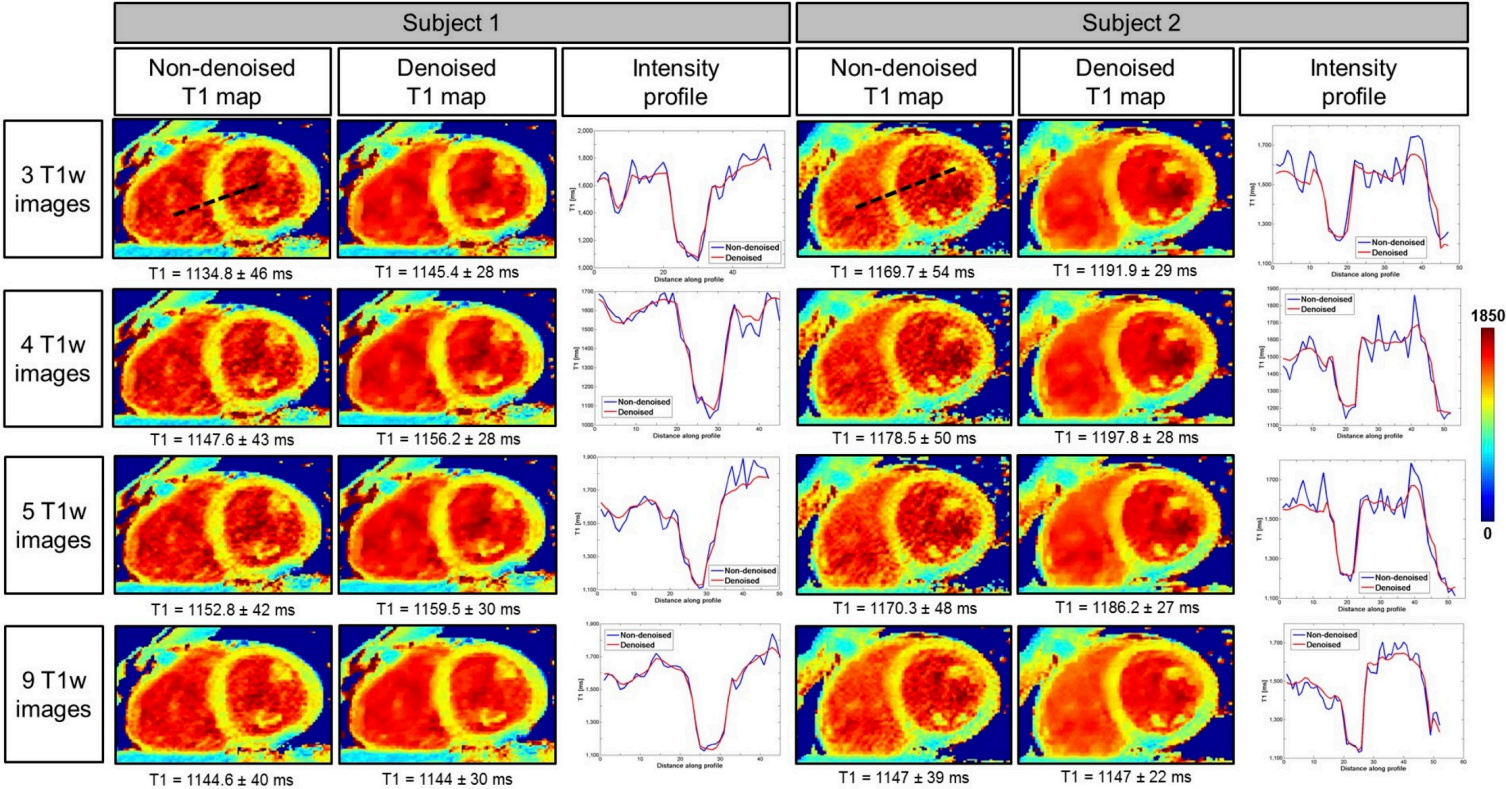
Myocardial 3D SASHA T1 maps of two representative healthy subjects before and after 3D denoising, with the accuracy and precision measured on the corresponding myocardial septum. T1 maps were retrospectively reconstructed by using respectively three, four, five and nine saturation time points along the recovery curve. For each T1 map, the intensity profiles across the blood and the myocardium are evaluated, and they are compared before (in blue) and after (in red) denoising.

3D SASHA T1 maps of two representative healthy subjects, obtained using a different number of saturation points (respectively three, four, five and nine points) before and after denoising are shown in Fig 6. The 3D denoising technique permits to recover the loss in precision due to the lower number of images along the recovery curve and to achieve comparable image quality between the T1 maps reconstructed with a different number of T1-weighted images, as confirmed by the intensity profiles across the blood and septum of the myocardium.

#### Prospective study

**Fig 7:**
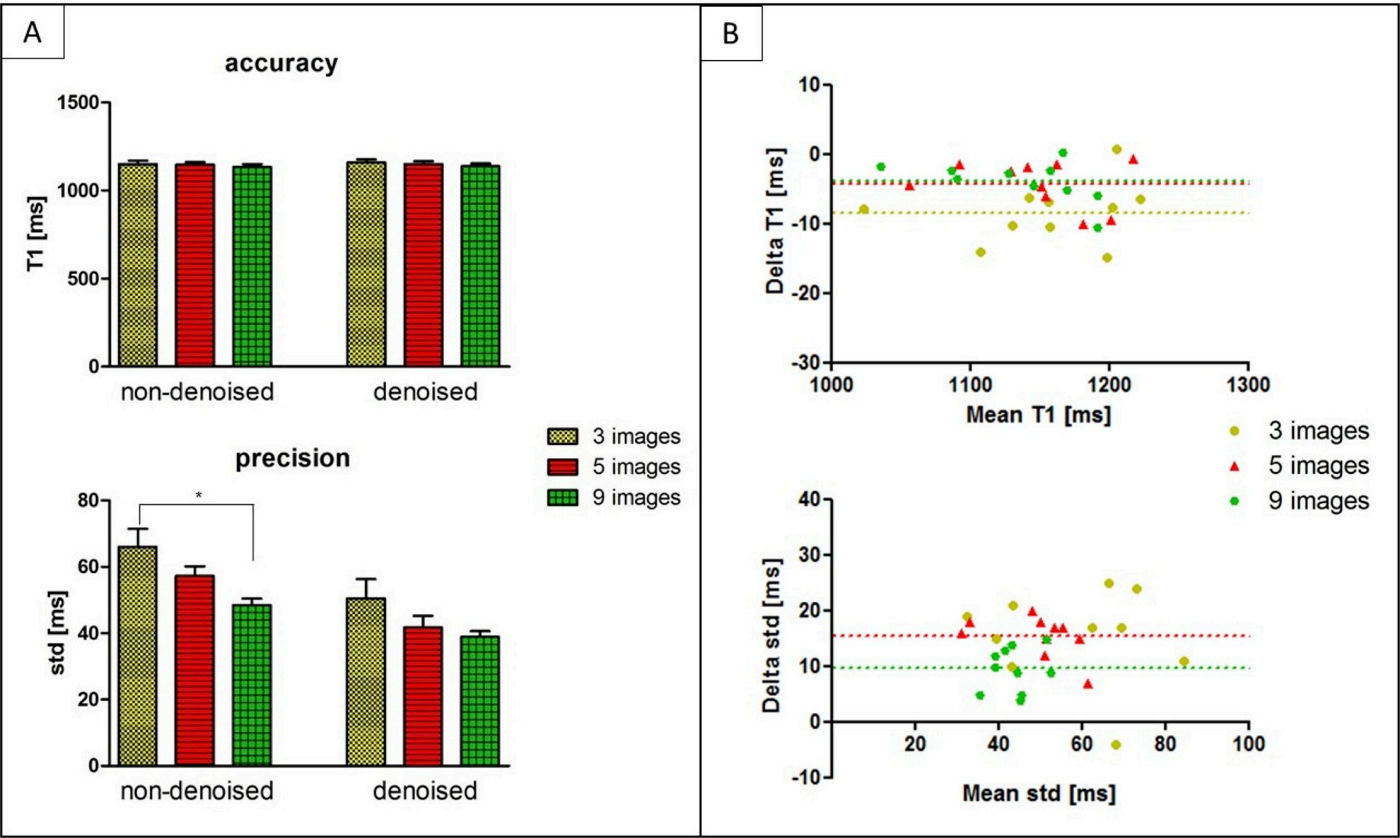
(A) Accuracy and precision averaged between the ten healthy subjects, acquired prospectively with 3D SASHA using three (yellow bar), five (red bar) and nine (green bars) saturation time points along the recovery curve. The measurements were performed before (non-denoised) and after (denoised) denoising in the mid slice in the septum of the myocardium. Statistical significant difference is indicated by * (p value < 0.034). (B) Bland Altman plot comparing the accuracy (on top) and precision (on bottom) of the 3D SASHA non-denoised vs. 3D SASHA denoised using three, five and nine T1-weighted images. Bias is reported for each graph. The x-axis of the plot shows the mean between the measurements of the non-denoised and denoised 3D SASHA, while the y-axis shows the difference (delta T1) between the two measurements.

The accuracy and precision averaged over all ten healthy subjects for the prospective study (three, five and nine T1-weighted images) both before and after denoising is shown in Fig 7A. There was no statistical difference between the accuracy measured on the T1 maps reconstructed using different number of T1-weighted images, both before (*P* = 0.73) and after (*P* = 0.64) denoising. However, a statistical difference (*P* < 0.05) was found between the precision measured on the T1 map acquired with three and nine T1-weighted images before denoising. After applying the 3D denoising technique, the precision was recovered with no statistical difference (*P* = 0.27) when three or nine images where used for T1 mapping. However, if five images were acquired instead of three, the precision improved by about 12%. The average scan time for the original 3D SASHA sequence (nine images along the recovery curve) was 12.9 ± 1.9 minutes. Scan time was reduced to 7.55 ± 0.7 and 5.6 ± 1.2 minutes when acquiring 5 and 3 images respectively. Fig 7B shows the Bland-Altman plots of the accuracy and precision of the 3D SASHA non-denoised vs. denoised for the three different acquisitions using three, five and nine T1-weighted images. The difference in terms of accuracy between non-denoised and denoised 3D SASHA is smaller when five and nine images are used. In terms of precision, the 3D denoising method has a major impact on the T1 maps acquired with three and five images, with a bias equal to 15.5 ms compared to 9.6 ms with nine images.

**Fig 8:**
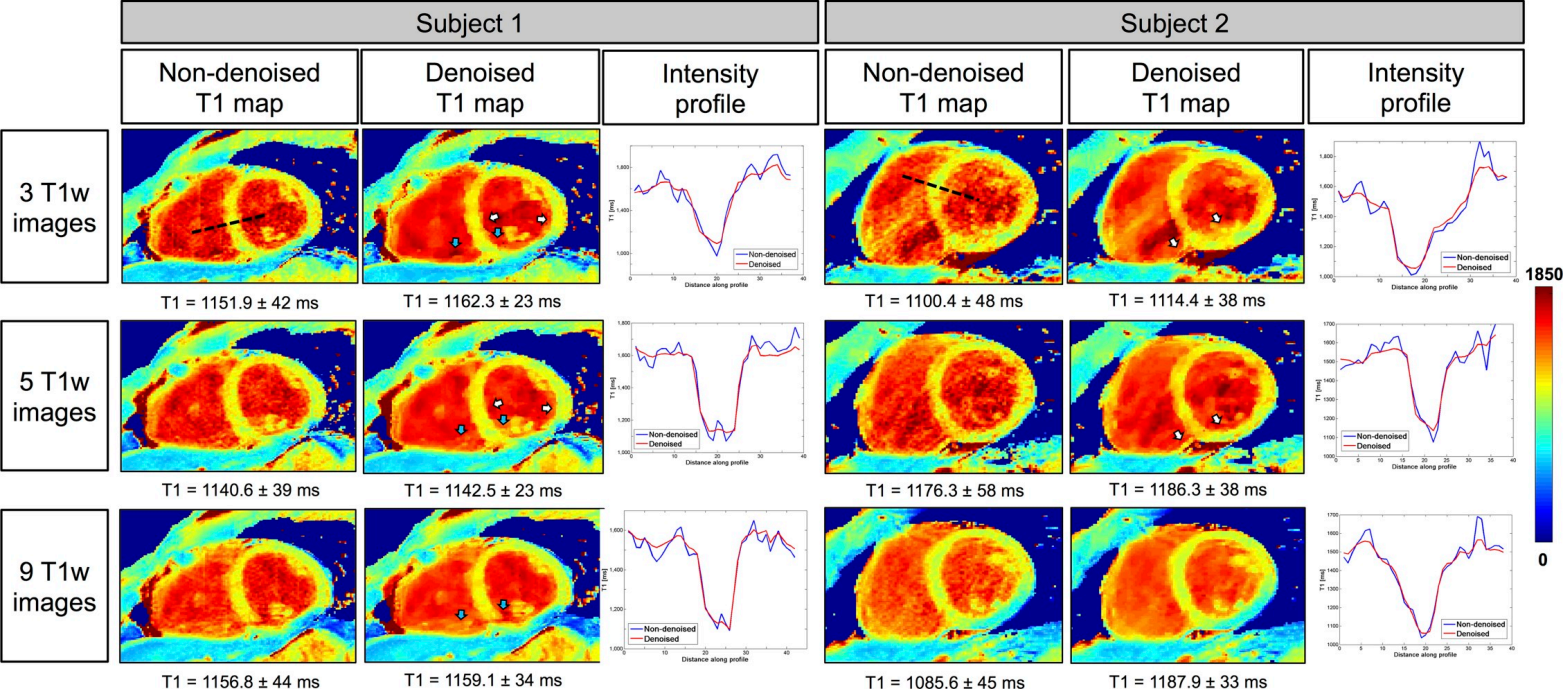
3D SASHA T1 maps of two representative subjects acquired prospectively with three, five and nine T1-weighted images along the saturation recovery curve, and the corresponding intensity profiles. The accuracy and precision measured in the myocardial septum are indicated for each T1 map. T1 maps were reconstructed before and after 3D denoising. There was an improvement in the image quality in terms of myocardial and papillary muscles delineation after 3D denoising (white arrows). Motion artefact (blue arrows) can be observed when more images are acquired due to the longer scan time. The intensity profiles across the blood and the myocardium, before (in blue) and after (in red) denoising, are shown for both subjects.

The 3D denoising technique permitted to preserve image quality of the 3D SASHA T1 maps when five T1–weighted images were acquired, as shown in Fig 8 in two representative healthy subjects for the prospective acquisition. This is also confirmed by the comparison of intensity profiles across the myocardium for the two subjects, shown in Fig 8. However, if only three T1-weighted images were acquired, the delineation of the myocardial borders and the papillary muscles was slightly degraded, as indicated by the white arrows. As the acquisition becomes longer with a higher number of saturation time points, it is also more prone to motion artefacts, as indicated by the blue arrows in the T1 maps for subject 1. S2 Fig shows the average image quality scores of the T1 maps acquired prospectively with three, five and nine T1-weighted images for all ten healthy subjects. There was no statistical difference (Kruskal-Wallis test) between the three different acquisitions. However, in general the T1 maps acquired using five T1-weighted images were found to have a slightly higher image quality score.

**Fig 9:**
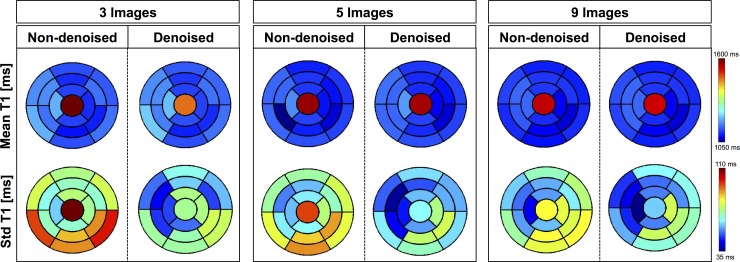
AHA plots for the accuracy (mean) and precision (standard deviation) of the 3D SASHA T1 maps from the ten healthy subjects of the prospective study. The T1 maps were acquired using three, five and nine T1-weighted images and they were obtained both before and after denoising. The accuracy is not affected after denoising, acquiring either 3, 5 or 9 images. Conversely, precision improves after denoising for the three different cases.

Fig 9 shows the AHA segmentation for the accuracy and precision measured on the 3D SASHA T1 maps acquired using three, five and nine T1-weighted images, reconstructed both before and after 3D denoising. The 3D denoising technique does not affect the accuracy of the 3D SASHA T1 maps for all the three different acquisitions (with three, five and nine T1-weighted images). The precision considerably improves after denoising across the whole left ventricle, particularly when three and five images are acquired.

### Patient study

**Fig 10:**
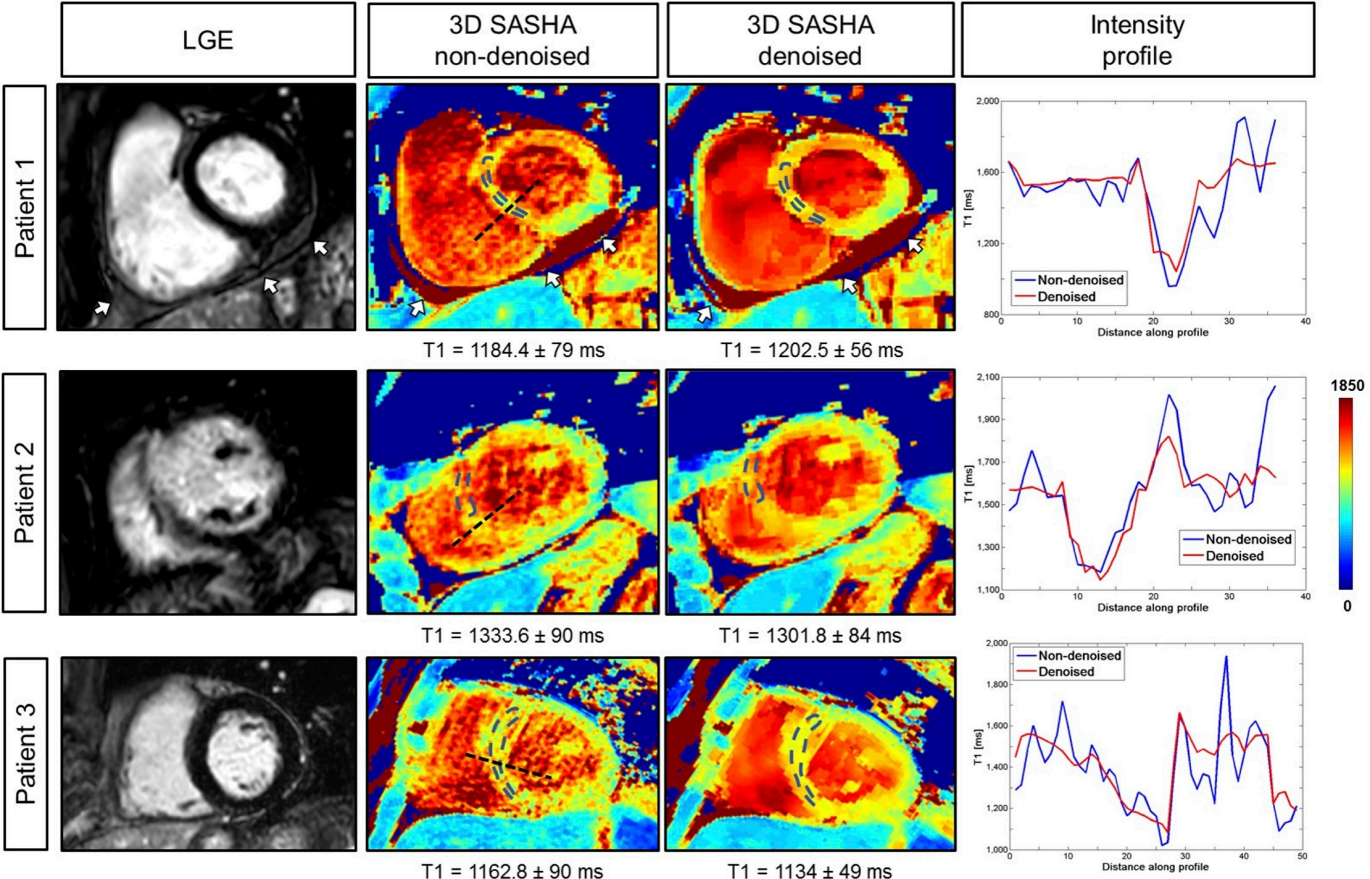
3D SASHA T1 maps before (non-denoised) and after (denoised) denoising and corresponding LGE image for three patients. For each patient, the intensity profiles across the blood and the myocardium of the T1 maps non-denoised (in blue) and denoised (in red) are shown. Patient 1 presented with pericarditis, indicated by the white arrow. Patient 2 had ischemia in the mid-apical septum-anterior wall with corresponding elevated myocardial T1 on the 3D SASHA T1 maps. Patient 3 did not present with disease, which was confirmed by the measured myocardial T1 values.

Native 3D SASHA T1 maps were obtained in patients using five saturation points prospectively acquired along the recovery curve. T1 maps of the three patients before and after denoising and the corresponding LGE images are shown in Fig 10. Patient 1 was diagnosed with pericarditis, which was detected from both the LGE and the 3D SASHA T1 mapping before and after denoising. The second patient has scar in the apical-mid anterior septum wall. There was an elevation of the T1 values corresponding to the myocardial scar tissue on the 3D SASHA T1 maps, which was in agreement with previously reported myocardial T1 values for scar tissue [23]. However, the septum in the 3D SASHA T1 map is not well delineated, probably due to residual respiratory and cardiac motion. The third patient, who did not show any cardiac disease, has T1 values in agreement with reference values for native pre-contrast healthy myocardial T1 [24]. There was no statistical difference regarding the accuracy and precision measured on non-denoised or denoised 3D SASHA T1 maps (respectively *P* = 0.91 and *P* = 0.19). The average scan time for the 3D SASHA sequence with five images along the recovery curve was about 8 minutes.

## Discussion

In this study, we proposed to accelerate the 3D SASHA myocardial T1 mapping technique by reducing the number of saturation time point images acquired along the recovery curve. Our retrospective experiments in a standardized phantom and healthy subjects demonstrate that reducing the number of T1-weighted images used for the T1 fitting has little impact on the accuracy of the T1 maps, independently on the denoising, as shown in Fig 2 and Fig 6. However, reducing the number of images has a direct impact on image quality and precision of the T1 maps. To recover the loss in precision and image quality of the myocardial T1 maps when a reduced number of T1-weighted images is acquired, we propose to employ a recently introduced 3D denoising technique based on the Beltrami regularization. The proposed approach was validated prospectively in healthy subjects and feasibility was shown in 3 patients with suspected cardiovascular disease.

The phantom and healthy subjects’ retrospective experiments demonstrated that the accuracy of 3D SASHA T1-mapping was not affected if the number of T1-weighted images considered for T1 mapping was reduced to three images. Moreover, the accuracy was not affected by the application of the 3D denoising technique. Conversely, the precision was reduced when a smaller number of T1-weighted images was employed, with a statistical difference between using three and nine images, whereas no statistical difference was observed after denoising. Image quality was affected by the number of time point images used for mapping (e.g. delineation of myocardial borders), which was improved after applying the proposed 3D denoising technique. Myocardial T1 values were in general lower in the infero-lateral wall, this has been observed in previous studies (10,) and may be due to residual motion and field inhomogeneity.

The prospective experiments in healthy subjects confirmed the results obtained in the initial retrospective study. The AHA segmentation confirmed that the accuracy is maintained despite the smaller number of T1-weighted images acquired when combined with the 3D denoising technique. The T1 variability was comparable to 2D MOLLI data previously published in the literature [12] when the 3D SASHA data was acquired with three and five T1-weighted images. The denoised 3D SASHA data acquired with nine T1-weighted images showed higher T1 variability within the left ventricle, probably due to residual respiratory motion. Although not statistically significant, the precision of the T1 map obtained from five T1-weighted images before denoising was about 12% higher than that of the T1 map obtained from three images. However, this improvement is based on a small cohort of healthy subjects, and further investigation in a larger cohort of subjects would be required to validate this finding. In terms of image quality, the delineation of the myocardial borders was in general worst in the T1 maps reconstructed from three images in comparison to the corresponding T1 maps reconstructed from five images (Fig 8). The quality of the T1 map depends highly on the quality of the T1-weighted images used for fitting. The T1-weighted image acquired at the shortest saturation time has usually low contrast and is more prone to motion related artefacts originating from e.g. chest wall, which can propagate into the T1 map. The latter has a stronger effect when only three T1-weighted images are acquired. By acquiring a larger number of T1-weighted images, the fit depends less on a single T1 weighted image, and consequently artifacts are less visible in the reconstructed T1 map. For the prospective study, some respiratory motion related artefacts were observed in the acquisitions with five and nine images consistent with the longer scan times. In general, a good compromise between image quality and motion related artefacts was observed when the T1 map was reconstructed using five images, as also confirmed by the image quality assessment.

Results from the patient study showed that the proposed accelerated 3D SASHA T1 mapping technique achieves good image quality and provides quantitative values consistent with the complementary LGE images and corresponding diagnosis. The proposed approach enabled a reduction in scan time of about 33% from ~12 minutes for the original 3D SASHA technique to approximately ~8 minutes for the proposed accelerated approach with five T1-weighted images along the recovery curve. Nevertheless, it was still dependent on the efficiency of the diaphragmatic navigator and its unpredictable scan time, which can severely drop in patients with irregular breathing patterns. Alternative motion compensation techniques, such as image-based navigation and self-navigation [25–28], will be investigated in future studies to achieve 100% respiratory scan efficiency and consequently to further accelerate the scan, which can provide more comfort to the patient and reduce the risk of introducing additional bulk motion artifacts associated with long scans.

The saturation time points were selected along the T1 recovery curve in order to provide an equal distribution between the shortest and the longest saturation time, following the original implementation of the 2D SASHA [4] imaging sequence. Further investigation is warranted to understand the effect of a different selection of the sampling points in terms of accuracy and precision of the T1 map [29].

The 3D Beltrami denoising technique employed in this study permits to considerably improve image quality and the precision of the 3D SASHA T1 maps, independently of the number of T1-weighted images acquired (ranging from nine to three). The denoising is a post-processing step and is applied to the T1-weighted images. Future studies will investigate the integration of the denoising technique as regularization directly in the reconstruction process to further accelerate the scan.

Due to time constraints, only native 3D SASHA T1-mapping was performed in this study. Further studies will investigate the acquisition of accelerated native and post-contrast 3D SASHA myocardial T1 maps in patients with cardiovascular disease to enable the measurement of extracellular volume fraction, which has been demonstrated to be useful to detect diffuse myocardial fibrosis [30]. Larger clinical studies are now warranted to validate the clinical value of the proposed imaging technique.

## Conclusions

In conclusion, we have demonstrated the feasibility of accelerating the free-breathing 3D SASHA T1 mapping imaging technique by reducing the number of T1-weighted images acquired along the saturation recovery magnetization evolution from nine to three. This has a little impact on the accuracy of the T1 map, while the expected loss in precision can be recovered with a 3D Beltrami denoising method. With five T1-weighted images a good compromise between scan time (8 minutes), image quality and precision can be achieved. The proposed approach has been validated in phantom, healthy subjects and patients with suspected cardiovascular disease.

## Supporting information

S1 Fig Bland-Altman plot of the accuracy of the non-denoised (blue) and denoised (red) 3D SASHA vs. the gold standard IRSE sequence, measured in the T1 phantom. (PDF)

S2 Fig Image quality assessment of the 3D SASHA T1 maps acquired using three, five and nine T1-weighted images, for the prospective study in healthy subjects. A 4-point scoring system was employed from 1: non-diagnostic images to 4: fully diagnostic images.
(TIFF)
