# Supplementary figures and images for: Faster 3D saturation-recovery based myocardial T1 mapping using a reduced number of saturation points and denoising
Source: PLoS One. 2020 Apr 10;15(4):e0221071. doi: 10.1371/journal.pone.0221071 (PMC7147792; doi:10.1371/journal.pone.0221071)

Supporting Information Figure S1:

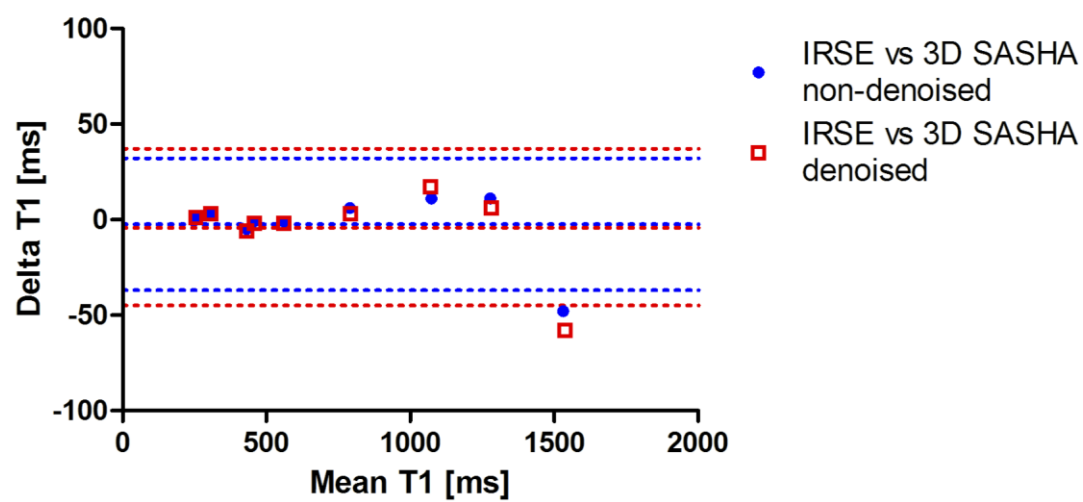

Supplement: S1 Fig — (PDF) [file pone.0221071.s001.pdf]

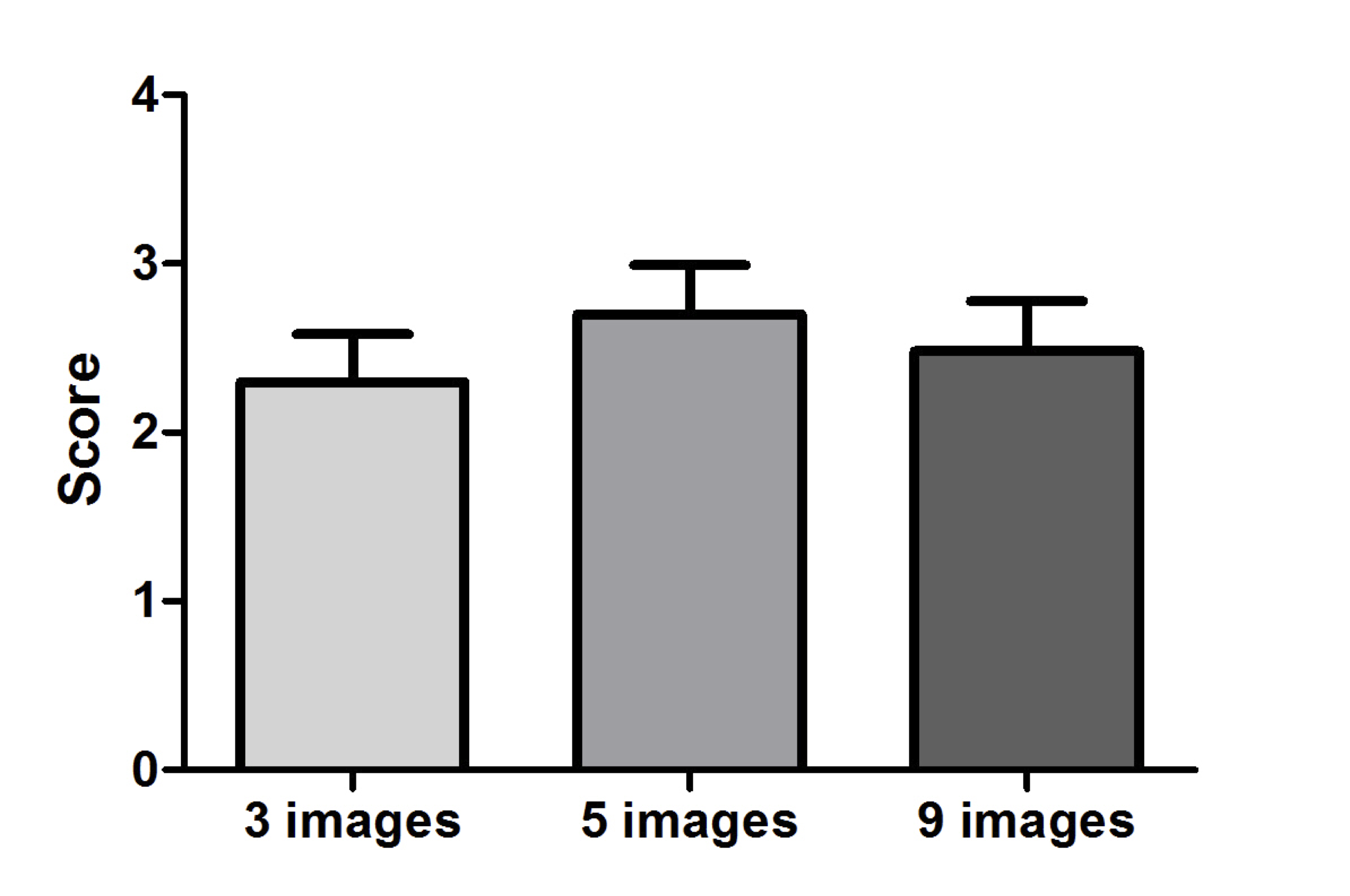

Supplement: S2 Fig — A 4-point scoring system was employed from 1: non-diagnostic images to 4: fully diagnostic images. (TIFF) [file pone.0221071.s002.tiff]
